# Co-localization of TAZ and H2A.Z up-regulates IL-6 expression in alveolar epithelial cells to polarize M2 cells to alleviate hypoxia-induced lung injury

**DOI:** 10.1016/j.gendis.2025.101713

**Published:** 2025-06-09

**Authors:** Paiyu Liu, Mengjie Zhang, Jitao Zeng, Yafei Gao, Xiaochong He, Wenying Liu, Peng Wang, Bing Ni

**Affiliations:** a Department of Pathophysiology, College of High Altitude Military Medicine, Army Medical University, Chongqing 400038, China; b Ultrasound Department, The 991th Hospital of the Joint Logistic Support Force of the People’s Liberation Army of China, Hubei 441000, China; c Reproductive Medical Center, Southwest Hospital, Army Medical University, Chongqing 400038, China; d Department of Nursing Administration, Faculty of Nursing, Army Medical University, Chongqing 400038, China

**Keywords:** Alveolar epithelial cells, H2A.Z, Hypoxia, IL-6, Lung injury, M2 macrophages, TAZ

## Abstract

Transcriptional co-activator with PDZ-binding motif (TAZ) plays a critical role in the repair process following lung injury by enhancing the proliferation of alveolar epithelial cells (AECs) and exerting anti-inflammatory effects post-injury. Hypoxia is a significant factor in inducing lung injury. However, whether and how TAZ regulates transcription of target genes involved in hypoxia-induced lung injury remains unclear. In this study, we discovered that M2 macrophages protected against hypoxic condition-induced lung injury, coinciding with increased TAZ expression in hypoxic AECs. Experimental verification confirmed that the up-regulation of TAZ promoted AEC proliferation. Furthermore, we found that TAZ interacted with H2A.Z, a histone H2A variant. CUT&Tag sequencing verified that TAZ was essential for maintaining the enrichment of H2A.Z at the promoters of their shared target genes in hypoxic AECs. Gene Ontology analysis revealed a significant predominance of pathways associated with IL-6 mediation, indicating that this pathway is the most enriched in our dataset. Importantly, IL-6 secreted by AECs under hypoxia stimulates the polarization of macrophages towards the M2 phenotype, thereby promoting lung tissue repair after hypoxia exposure. In summary, our findings demonstrate that TAZ facilitates the deposition of H2A.Z at the IL-6 gene promoter, thereby enhancing IL-6 expression in hypoxic AECs. This, in turn, induces M2 macrophage polarization and PD-L1 expression, ultimately mitigating hypoxic condition-induced lung injury.

## Introduction

The lung is a vital organ within the human respiratory system, essential for gas exchange and the proper functioning of numerous physiological processes. However, factors such as high-altitude hypoxia, viral infections, and smoking frequently cause damage to lung tissue, resulting in diminished lung function and potentially progressing to chronic obstructive pulmonary disease or acute respiratory distress syndrome, thereby posing a significant threat to patients’ quality of life and safety.^1^, ^2^, ^3^ Inadequate oxygen supply is considered a significant factor contributing to lung damage and has been documented to induce inflammatory cell infiltration, thickening of the alveolar septa, and a reduction in alveolar size within lung tissue.^2^^,^^4^ Currently, there are no effective solutions to reverse hypoxia-induced lung damage, which underscores the critical importance of understanding the body’s repair mechanisms.

Following lung injury, the self-repair process is critical for maintaining respiratory function, preventing infections, mitigating inflammation, and avoiding fibrosis.^5^ This complex process involves the coordinated actions of various cell types and the precise regulation of molecular mechanisms.^5^^,^^6^ Alveolar epithelial cells (AECs) play a central role in this repair process by maintaining alveolar structural integrity and modulating inflammatory responses.^6^^,^^7^ Furthermore, studies have demonstrated that in lipopolysaccharide-induced lung injury models, dexmedetomidine reduces the activation of NLRP3 inflammasomes in the lung epithelium and promotes the polarization of alveolar macrophages towards the M2 phenotype, which is essential for lung injury repair.^8^ M2 macrophages are pivotal in resolving inflammation and facilitating tissue remodeling and repair.^9^ However, the mechanisms by which AECs modulate macrophage polarization under conditions of high-altitude hypoxia to alter the local immune microenvironment and enhance repair remain poorly understood.

Several studies have demonstrated that epigenetic regulatory mechanisms, such as histone modifications, DNA methylation, and non-coding RNA regulation, play a pivotal role in the repair of lung injuries.^10^, ^11^, ^12^ These mechanisms precisely modulate the function of AECs and the behavior of immune cells by influencing gene expression patterns.^11^^,^^13^ Consequently, a comprehensive understanding of the repair mechanisms following lung injury, particularly the interplay between AECs and immune cells, and the significance of epigenetic regulation, is crucial for the development of novel therapeutic strategies.

The role of the transcriptional co-activator with PDZ-binding motif (TAZ) is garnering increasing attention. Recent studies have demonstrated that under hypoxic conditions, the protein expression level of TAZ markedly elevates.^14^^,^^15^ Furthermore, an expanding body of evidence indicates that TAZ protein in AECs plays a critical role in the repair process following lung injury, not only by exerting anti-inflammatory effects post-injury but also by promoting the proliferation and differentiation of AECs.^16^, ^17^, ^18^ This highlights its potential significance in cellular adaptation to hypoxic environments and the facilitation of damage repair. Additionally, the interaction between TAZ and chromatin remodeling complexes, such as sucrose nonfermenting 2-related CREB binding protein activator protein (SRCAP), influences the deposition of H2A.Z at target gene loci.^19^ H2A.Z, a variant of histone H2A, plays a vital role in regulating transcription, cell cycle control, DNA replication, and other cellular processes.^20^ Research has shown that the deposition of H2A.Z on chromatin is essential for transcriptional activity.^20^^,^^21^ However, these findings have not been thoroughly investigated under hypoxic conditions. Moreover, our previous study with the bait protein–protein interaction-sequencing (bPPI-seq) strategy indicated that H2A.Z may interact with TAZ in 293T cells.^22^ Nevertheless, whether TAZ interacts with H2A.Z and consequently plays a pivotal role in the pathogenesis of hypoxia-induced lung injury remains to be elucidated.

In this study, we investigate the levels of TAZ and H2A.Z deposition at target genes in hypoxic AECs using cleavage under targets and tagmentation (CUT&Tag) sequencing. Our findings indicate that TAZ facilitates the deposition of H2A.Z on the promoters of target genes, thereby regulating transcription under hypoxic conditions. The interaction between TAZ and H2A.Z induces the secretion of interleukin (IL)-6 in hypoxic AECs, which in turn promotes the polarization of macrophages toward the M2 phenotype, a critical process for lung tissue repair. Consequently, our research identifies TAZ in AECs as a pivotal regulatory factor influencing the positioning of H2A.Z on target genes, potentially offering novel insights into the mechanisms of lung injury repair.

## Material and methods

### Cell culture and treatment

TC-1 cells (Bluefcell Biotechnology, BFN 60805941), RAW264.7 cells (Bluefcell Biotechnology, BFN607200597), and bone marrow-derived macrophages (BMDM) were cultured in Dulbecco’s modified Eagle medium (DMEM) (GIBCO, C11995500BT) supplemented with 10% fetal bovine serum (Umedium, 3022A) and 1% penicillin/streptomycin mixture (biosharp, BL505A). Cells were incubated at 37 °C in a humidified atmosphere containing 5% CO_2_.

### Mice

Male C57BL/6J wild-type mice, aged 8–12 weeks and weighing 22–26 g, were purchased from the Experimental Animal Center of the Army Medical University in Chongqing, China. To establish an acute hypoxia-induced lung injury mouse model, mice were randomly assigned to normoxic and hypoxic groups. The normoxic group was maintained at the Experimental Animal Center of the Army Medical University. In contrast, the hypoxic group was placed in a simulated low-pressure chamber at the High Altitude Medicine Department of the Army Medical University, simulating an altitude of 5800 m. Both groups were housed for 4 days and 14 days before being anesthetized with sevoflurane, after which bronchoalveolar lavage fluid (BALF) and lung tissue samples were collected for analysis. To investigate the role of M2 macrophages in hypoxia-induced lung injury, M2-polarized BMDM were administered via tail vein injection (1 × 10^6^ cells per mouse) or an equivalent volume of phosphate-buffered saline (PBS). A subset of mice was housed at the Experimental Animal Center of the Army Medical University, while others were placed in the simulated low-pressure chamber at the High Altitude Medicine Department. Four days later, mice were anesthetized with sevoflurane, and BALF and lung tissue samples were collected for analysis. The experimental protocol was approved by the Animal Welfare and Ethics Committee of the Army Medical University.

### Isolation and alternative activation of BMDM

C57 BL/6 mice were euthanized, and their femurs and tibias were extracted and disinfected with 75% alcohol for 5 min. Bone marrow cells were flushed out and cultured in DMEM (GIBCO, C11995500BT) containing 20 ng/mL of recombinant mouse macrophage colony-stimulating factor (PeproTech, 315-02-10UG). After 3 days, the culture was supplemented with macrophage colony-stimulating factor-containing DMEM and continued to be culture. On day 7, the BMDM were considered M0 macrophages. Subsequently, M0 macrophages were stimulated with 20 ng/mL IL-4 (PeproTech, 214-14) for 24 h to induce M2 polarization.

### Co-culture system of AECs and macrophages

To establish the co-culture model, TC-1 cells (2.5 × 10^5^ cells/well) were seeded in the upper compartment (transwell) of a transwell plate (Merck Millipore, MCHT12H48). Concurrently, RAW264.7 cells (2.5 × 10^5^ cells/well) were seeded in the lower compartment of the plate. After adding the appropriate culture medium, the co-culture system was incubated in a cell culture incubator at 37 °C with 5% CO_2_.

### Quantitative reverse transcription PCR

Total RNA was extracted from cells lysed with Trizol (ABclonal, RM30129). Reverse transcription was performed using the ABScriPT Neo RT Master Mix for qPCR with gDNA Remover (ABclonal, RK20433). Quantitative reverse transcription PCR was conducted using the 2X Universal SYBR Green Fast qPCR Mix (ABclonal, RM21203) on a real-time PCR system (Bio-Rad). Primer sequences were as follows: WWTR1-F: CCTCAGCAACATGGACGAGA; WWTR1-R: CAGACTCCAAAGTCCCGAGG; IL-6-F: GAGAGGAGACTTCACAGAGGATACC; IL-6-R: TCATTTCCACGATTTCCCAGAGAAC.

### Cell tracing assay

Cell tracing was performed using the CFDA SE Tracing Assay Kit (Beyotime, C0051). Cells were labeled with the provided cell labeling solution and storage solution at 37 °C for 10 min, followed by the addition of 10 mL of complete cell culture medium. After gentle mixing at room temperature, cells were centrifuged, and the supernatant was discarded. Subsequently, 10 mL of complete cell culture medium was added, and cells were incubated at 37 °C for 5 min. Labeled cells were then ready for transplantation into mice.

### Immunofluorescence assay

Cell samples were fixed with 4% paraformaldehyde for 15 min and permeabilized with 0.1% Triton X-100 for 15 min. After washing, they were incubated with 3% bovine serum albumin (Beyotime, ST023) at room temperature for 30 min. The samples were then incubated with the primary antibodies (diluted in PBS) at 4 °C overnight. TAZ (Invitrogen, PA1-46190), H2A.Z (Abcam, Ab150402), surfactant protein C (Sp-C; Abcam, Ab211326), and homeodomain-only protein homeobox (HOPX; ABclonal, A15537PM) were used as primary antibodies. After washing with PBS supplemented with Tween-20 three times for 5 min each, the samples were incubated with horseradish peroxidase-polymer anti-rabbit secondary antibodies from the Double Staining Three-Color Multiple Fluorescence Staining Kit (AiFang Biological, AFIHC023) at room temperature for 30 min. TYR-570 and TYR-520 fluorescent dyes were applied for 5 min each, followed by washing with PBS supplemented with Tween-20 three times for 5 min each. 4′,6-diamidino-2-phenylindole (DAPI; Servicebiom, G1012) was used for nuclear staining at room temperature in the dark for 10 min. After washing with PBS three times, an anti-fluorescence quenching sealing agent was applied for sealing.

### Flow cytometry analysis

The cells were scraped and transferred into a 1.5 mL tube. The cells were resuspended in culture medium and adjusted to a concentration of 1 × 10^6^ cells/mL. Cytometry antibodies, including CD206 (Biolegend, 141719), F4/80 (Biolegend, 123107), and CD11b (Biolegend, 101208), were added. The cells were incubated at room temperature in the dark for 40 min. Finally, the cells were resuspended in PBS and analyzed using a flow cytometer.

### Hematoxylin and eosin staining

Lung tissue samples were embedded in paraffin and sectioned. The sections were stained with hematoxylin and eosin staining solutions. The staining procedure involved incubating the sections in hematoxylin solution for 5 min, followed by eosin solution for 3 min. After staining, the sections were digitally scanned using a PreciPoint M8 scanner and analyzed according to the Smith scoring system.^23^

### Western blotting assay

Total protein was extracted using RIPA lysis buffer (Beyotime Biotechnology, P0013B) containing protease inhibitors (ABclonal, RM02916), phosphatase inhibitors (Beyotime Biotechnology, P1045), and the serine protease inhibitor phenylmethanesulfonyl fluoride (PMSF; Beyotime Biotechnology, ST505). Protein concentration was determined using the bicinchoninic acid assay kit (Beyotime Bio, P0009). Proteins were mixed with loading buffer and heated at 100 °C for 5 min, then separated on SDS-PAGE according to their molecular weight, and transferred onto PVDF membranes (Millipore, IPVH00010). The membranes were blocked with 5% skim milk at room temperature for 2 h and incubated at 4 °C overnight with the following primary antibodies: inducible nitric oxide synthase (iNOS) antibody (Abcam, ab189745), arginase 1 (ARG1) antibody (Abcam, ab203490), TAZ antibody (Invitrogen, PA1-46190), H2A.Z antibody (Abcam, Ab150402), β-actin antibody (Servicebio, GB15001-100), IL6 antibody (Abcam, ab259341), signal transducer and activator of transcription 3 (STAT3) antibody (MedChemExpress, HY-P80344), Phospho-STAT3 antibody (MedChemExpress, HY-P80857), and programmed cell death ligand 1 (PD-L1) antibody (Proteintech, 66248-1-IG). The membranes were then incubated with horseradish peroxidase-conjugated goat anti-rabbit IgG (Proteintech, SA00001-2) at room temperature for 1 h and visualized using enhanced chemiluminescence on the ChemiDocTM Imaging System (Bio-Rad). Bands were analyzed using Image J.

### Immunohistochemistry

Immunohistochemical staining was performed on paraffin-embedded lung tissues. Slides were treated with an endogenous peroxidase blocking solution (Beyotime, P0100B) for 30 min, followed by three washes with PBS supplemented with Tween-20 for 5 min each. A 5% bovine serum albumin blocking buffer was applied, and slides were incubated at room temperature for 1 h. After discarding the blocking solution, TAZ antibody (Invitrogen, PA1-46190) was added and incubated at 4 °C overnight. Slides were allowed to warm to room temperature for 1 h, and then washed. Peroxidase-labeled secondary antibodies (Beyotime, A0208) were applied and incubated at room temperature for 30 min. After washing, 3,3′-diaminobenzidine chromogen solution (AiFang biological, AFIHC004) was added, followed by counterstaining with hematoxylin (Beyotime, C0107), and the slides were observed under a microscope. After staining, the sections were digitally scanned using a PreciPoint M8 scanner and analyzed.

### Stable knockdown cells

Lentiviral particles for TAZ knockdown were sourced from GeneChem Company. TC-1 cells were transduced with lentiviral supernatants containing shRNA targeting TAZ. Following 48-h incubation, the cells were selected with 2 mg/mL puromycin to enrich for infected cells. The targeting sequence for TAZ knockdown was CCATGAGCACAGATATGAGAT.

### Cell viability assay

Cells were seeded at a density of 2000 cells per well in a 96-well plate. At the time of assessment, the original culture medium was removed, and 100 μL of DMEM culture medium containing 10% CCK-8 solution (BIOGROUND, BG0025) was added to each well. Following a further incubation at 37 °C for 2 h, the absorbance of each well was measured at 450 nm using a microplate reader.

### EdU (5-ethynyl-2′-deoxyuridine) assay

A cell proliferation detection kit (EdU) (Beyotime, C0075L) was utilized to determine cell proliferation. Cells were seeded at a density of 5800 cells per well in 48-well plates and incubated with the EdU working solution at 37 °C for 2 h after attachment. The cells were then fixed with 4% paraformaldehyde at room temperature for 15 min. After washing, 0.3% Triton X-100 was added and incubated at room temperature for an additional 15 min. Following further washes, the Click reaction mixture containing Azide 555 was added and incubated in the dark at room temperature for 30 min. Cell nuclei were counterstained with Hoechst 33342. After completion of the fluorescence detection, analysis was conducted using Image J software.

### Co-immunoprecipitation (Co-IP)

Cell lysates were extracted and incubated with Protein AG Magnetic Beads (Abbkine, KTI1010-CN) from the co-immunoprecipitation toolkit and primary antibody solution or IgG negative control at 4 °C overnight. The beads were then resuspended in 1 × wash buffer three times, followed by the addition of 1 × SDS-PAGE loading buffer and heating at 100 °C for 6 min. Western blotting was performed for detection and analysis.

### CUT&Tag experiment

The CUT&Tag experiment was performed according to the manufacturer’s protocol for the TD 903 kit (Vazyme, TD 903-02). Cells were collected at room temperature, washed, and centrifuged. After removing the supernatant, cells were resuspended in 100 μL of wash buffer. Subsequently, cells were transferred to centrifuge tubes preloaded with activated ConA beads and incubated at room temperature for 10 min. Following brief centrifugation, the tubes were placed on a magnetic rack, and the supernatant was carefully removed once it became clear. Primary and secondary antibody incubation steps were then conducted. After incubation, samples were centrifuged again, and the supernatant was cleared using a magnetic rack. To each sample, 2 μL of pA/G-Tnp and 98 μL of Dig-300 buffer were added, ensuring thorough mixing of the transposase with the cell (and nucleus)-bead complexes by gentle tapping. The samples were incubated at room temperature for 1 h with rotation. After incubation, samples were centrifuged, and the supernatant was cleared using a magnetic rack. Then, 40 μL of Dig-300 buffer and 10 μL of 5 × concentrated TTBL were added to each sample. These mixtures were transferred to 8-tube strips (Servicebio, PCR-0802-FC) and incubated in a PCR machine at 37 °C for 60 min. Afterward, 5 μL of proteinase K and 100 μL of buffer L/B were added to the samples, along with 20 μL of DNA extraction beads, mixed thoroughly, and incubated in a 55 °C metal bath for 10 min, gently shaking every 2 min. Beads were washed with buffer WA and buffer WB, and then placed on a magnetic rack to dry for 5 min until the beads' surface was dry. Finally, 16 μL of sterile water (ddH_2_O) was added for DNA elution. For library construction, 15 μL of the eluate was added to 8-tube strips containing indexed primers and 25 μL of 2 × CAM buffer. Samples were amplified in a PCR machine. Following amplification, VAHTS DNA Clean Beads (Vazyme, N411-02) were used to purify the library. The purified library was then sequenced to assess the experimental results.

### ChIP-quantitative PCR assay

A final concentration of 1% formaldehyde solution was added to the cell culture medium and incubated at 37 °C for 8 min, followed by the addition of Glycine Solution and incubation at room temperature for 5 min. Cells were washed twice with ice-cold PBS (containing protease inhibitors, PMSF, ABclonal, RM02916) and collected into centrifuge tubes. Cells were centrifuged at 4 °C at 1000g for 2 min. Using the chromatin immunoprecipitation (ChIP) assay kit (P2078), cells were resuspended in 200 μL of SDS lysis buffer (containing PMSF), lysed on ice for 10 min, and then sonicated. The samples were then centrifuged at 4 °C at 12000 *g* for 8 min. Supernatants were transferred to new EP tubes with the addition of 1.8 mL of ChIP dilution buffer (containing PMSF). Twenty microliters were taken as Input, and 200 μL were added with IgG (as a negative control). The remaining supernatant was divided into two portions, each with the addition of 2 μL of TAZ antibody (Invitrogen, PA1-46190) and H2A.Z antibody (Abcam, Ab150402) as experimental groups, and incubated at 4 °C overnight. Protein A + G from the ChIP assay kit was added to each sample (60 μL) and rotated at 4 °C for 60 min. Samples were centrifuged at 4 °C at 1000 *g* for 2 min, and the supernatant was discarded. Pellets were washed with wash solutions from the ChIP assay kit. Elution buffer (500 μL) was added for elution, samples were centrifuged, and the supernatant was transferred to new EP tubes. All sample supernatants and Inputs were heated at 65 °C for 4 h. Then, 2 μL of 20 mg/mL proteinase K was added to the supernatant and incubated at 45 °C for 60 min. For quantitative reverse transcription PCR analysis of *IL6*, the following primers were used: forward, 5′-CTGCTCACTTGCCGGTTTTC-3′; reverse, 5′-AGCATCAGTCCCAAGAAGGC-3′.

### Enzyme-linked immunosorbent assay (ELISA)

The supernatant was collected for measurement while the cells in the 6-well plate were counted. For the ELISA assay (ABclonal, RK00008), 100 μL of the prepared biotinylated antibody solution (ABclonal, RK00008) was added to each well and incubated at 37 °C for 1 h. Subsequently, 100 μL of streptavidin-horseradish peroxidase solution (ABclonal, RK00008) was added to each well. After washing the plate, 100 μL of 3,3′,5,5′-tetramethylbenzidine (TMB; ABclonal, RK00008) substrate solution was added to each well and incubated in the dark for 15 min. Upon observing a color change, 50 μL of stop solution was immediately added to each well. Finally, the absorbance of each well was measured at 450 nm using a microplate reader. The obtained values were subjected to further analysis.

### Statistical analysis

Data analysis was performed using GraphPad Prism 8. All data were presented as mean ± standard error of the mean with a 95% confidence interval. Student’s *t*-test was used to compare the two groups. One-way ANOVA was used to analyze differences among three or four groups, followed by Tukey’s multiple comparison test. ∗∗*P* < 0.01 and ∗*P* < 0.05 were considered statistically significant.

## Results

### M2 macrophages play a protective role against hypoxia-induced lung injury

**Figure 1:**
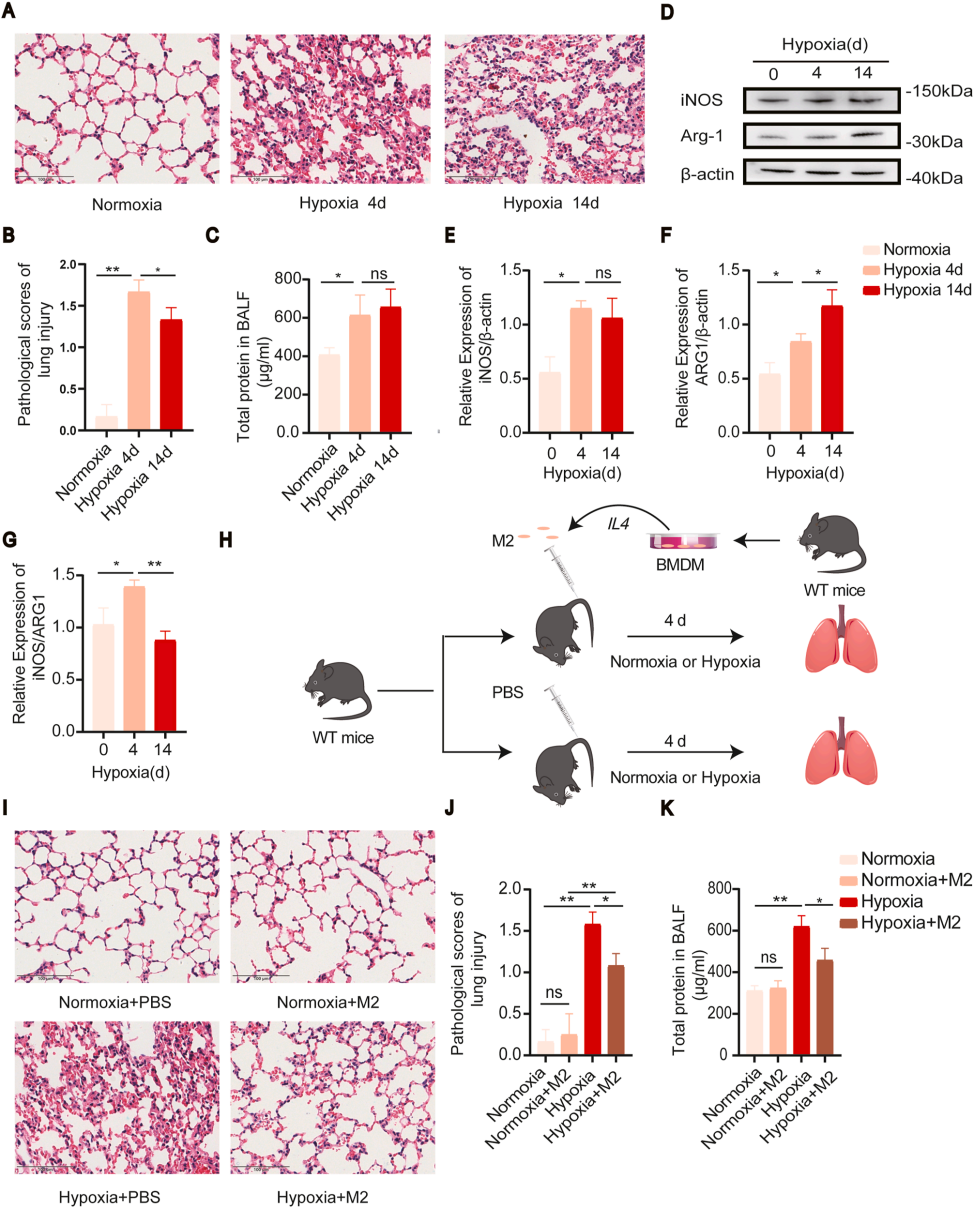
M2 macrophages play a protective role against hypoxia-induced lung injury. **(A)** Hematoxylin-eosin staining of mouse lung tissues at various time points under hypoxia. Scale bar = 100 μm. **(B)** Lung injury scores. *n* = 9. **(C)** Analysis of total protein concentration in BALF. *n* = 9. **(D**–**G)** Western blotting analysis and quantification of the expression of iNOS (an M1 marker) and Arg-1 (an M2 marker) in mouse lung tissues. β-actin served as a loading control. *n* = 3. **(H)** Schematic representation of the macrophage adoptive transfer experimental protocol. **(I)** Representative hematoxylin-eosin staining of mouse lung tissues. Scale bar = 100 μm. **(J)** Lung injury scores. *n* = 6. **(K)** Analysis of total protein concentration in BALF. *n* = 6. BALF, bronchoalveolar lavage fluid; iNOS, inducible nitric oxide synthase; Arg-1, arginase 1.

To evaluate the impact of hypoxia on lung tissue pathology in mice, we developed a hypoxia model by simulating an oxygen environment at an altitude of 5800 m above sea level using a high-altitude hypoxia chamber. Mice were housed under hypoxic conditions for 4 days and 14 days. Histological analysis of lung tissue sections stained with hematoxylin and eosin revealed significant pathological changes in mice exposed to hypoxia for 4 days compared with those under normoxic conditions. These changes included alveolar collapse, septal thickening, and increased infiltration of inflammatory cells (Fig. 1A and B). Furthermore, the bicinchoninic acid assay of BALF indicated elevated total protein concentrations in mice subjected to 4 days of hypoxia (Fig. 1C). Notably, after 14 days of hypoxia, some pathological changes in the lung tissue were partially alleviated, as evidenced by a reduction in inflammatory cell infiltration (Fig. 1A and B). However, the total protein concentration in BALF did not show a significant difference between the 4-day and 14-day hypoxic groups (Fig. 1C). These findings suggest that acute hypoxia can induce lung injury, but the body may develop adaptive mechanisms over time to mitigate the progression of lung damage.

Previous research has demonstrated that M2 macrophages can modulate the alterations in the lung microenvironment induced by hypoxia, thereby restoring lung immune function.^24^ In our study, we investigated the changes in macrophage polarization levels under normoxic, 4-day hypoxic, and 14-day hypoxic conditions. Western blotting analysis of lung tissue revealed an increased expression of the M1 macrophage marker iNOS after 4 days of hypoxia compared with normoxic conditions, with no significant difference in iNOS expression between 4 days and 14 days of hypoxia (Fig. 1D and E). Conversely, the M2 macrophage marker ARG1 exhibited increased expression on day 14 of hypoxia (Fig. 1D, F). Additionally, the iNOS/ARG1 ratio reached its peak at 4 days and subsequently decreased to control levels (Fig. 1D, G). These findings suggest that M1 macrophage activation may be the primary driver of increased lung injury under acute hypoxic conditions. However, the up-regulation of M2 macrophages during prolonged hypoxia may contribute to the regulation of the lung microenvironment, attenuation of inflammatory responses, and adaptation to hypoxia-induced lung injury.

Subsequently, we systematically assessed the role of M2 macrophages in the repair of lung injury. BMDMs were isolated from mice and induced to differentiate into M2 macrophages, which were subsequently validated using flow cytometry (Fig. S1A). The M2 macrophages were labeled with CFDA SE dye, and their presence in the lungs was confirmed via fluorescent imaging (Fig. S1B). The mice were then placed in a hypoxia chamber for 4 days to induce an acute hypoxia model. Histological examination of lung tissue sections stained with hematoxylin and eosin demonstrated that lung injury was significantly mitigated in mice treated with M2 cells (Fig. 1I and J). Specifically, compared with mice exposed only to hypoxia, those receiving M2 cell injections exhibited reduced alveolar collapse, decreased septal thickness, and diminished inflammatory cell infiltration and red blood cell aggregation (Fig. 1I). Furthermore, the total protein concentration in BALF from M2 cell-injected mice was lower (Fig. 1K). Under normoxic conditions, no significant differences in lung tissue pathology or BALF protein concentrations were observed, irrespective of M2 macrophage administration (Fig. 1I–K). These results suggest that M2 macrophages exert a protective effect against hypoxia-induced lung injury.

### Hypoxia up-regulates TAZ expression in AECs

**Figure 2:**
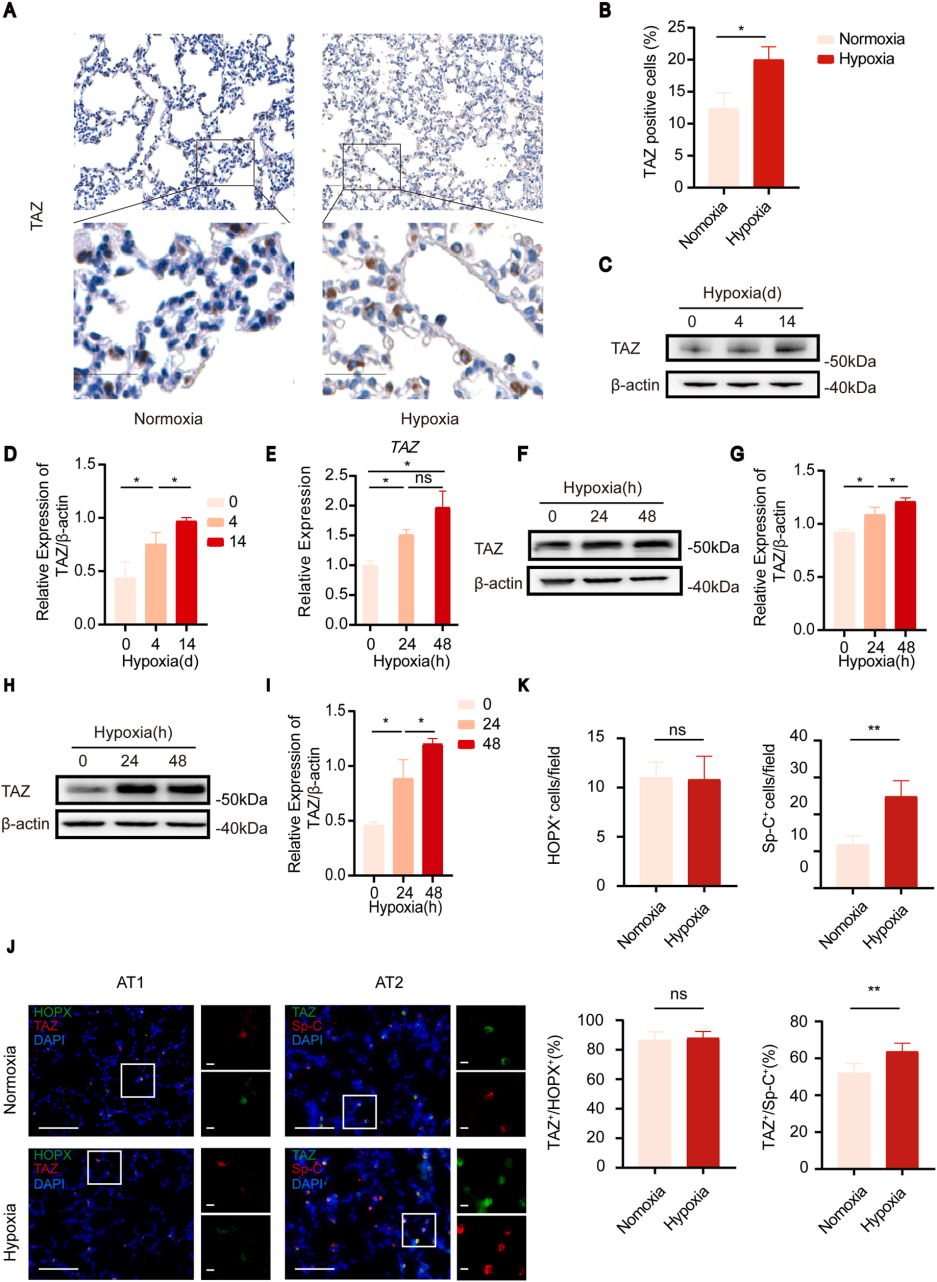
Hypoxia up-regulates TAZ expression in AECs. **(A)** Immunohistochemical results of lung tissue sections demonstrating the expression of TAZ. Scale bar = 20 μm. *n* = 5. **(B)** The bar graph illustrating the quantitative evaluation of TAZ expression in representative sections. **(C, D)** Western blotting analysis and quantification of TAZ expression in mouse lung tissues. β-actin was used as a loading control. *n* = 3. **(E)** Relative mRNA expression of *TAZ* in the alveolar epithelial cell line TC-1. *n* = 3. **(F, G)** Western blotting analysis and quantification of TAZ expression in the mouse alveolar epithelial cell line TC-1. β-actin was used as a loading control. *n* = 3. **(H, I)** Western blotting analysis and quantification of TAZ expression in the mouse alveolar epithelial cell line MLE12. β-actin was used as a loading control. *n* = 3. **(J, K)** Immunofluorescence staining was used to detect the number and proportion of TAZ^+^ cells in AT1 (HOPX^+^) and AT2 (Sp-C^+^) cells in lung tissue. *n* = 5. TAZ, transcriptional co-activator with PDZ-binding motif; AEC, alveolar epithelial cell.

To ascertain the alterations in TAZ expression levels in the lungs of mice subjected to acute hypoxia, we conducted immunohistochemical staining and Western blotting analysis on lung tissues, which revealed elevated TAZ expression in AECs of hypoxic mice (Fig. 2A–D). Subsequently, we utilized a hypoxia workstation to mimic hypoxic conditions (1% O_2_, 5% CO_2_, and 94% N_2_) for culturing AECs and assessed changes in TAZ expression levels in the TC-1 cell line through quantitative PCR and Western blotting. The findings indicated a substantial increase in TAZ protein expression at 24 h and 48 h post-hypoxia exposure relative to the control group (Fig. 2E–G). Comparable outcomes were observed in an additional mouse alveolar epithelial cell line, MLE12 (Fig. 2H and I), suggesting that TAZ expression in AECs is markedly induced following hypoxia.

We further investigated whether TAZ was expressed in HOPX^+^ type I alveolar epithelial cells (AT1) cells and Sp-C^+^ type II alveolar epithelial cells (AT2) cells under normoxic and hypoxic conditions using an immunofluorescence assay on mouse lung tissue sections, respectively. It was observed that the number of AT2 cells significantly increased following hypoxia exposure, whereas the number of AT1 cells remained unchanged. Additionally, the proportion of TAZ^+^ AT2 cells exhibited a marked increase, while no significant difference was detected in the proportion of TAZ^+^ AT1 cells (Fig. 2J and K).

### Hypoxia promotes AEC proliferation through TAZ

**Figure 3:**
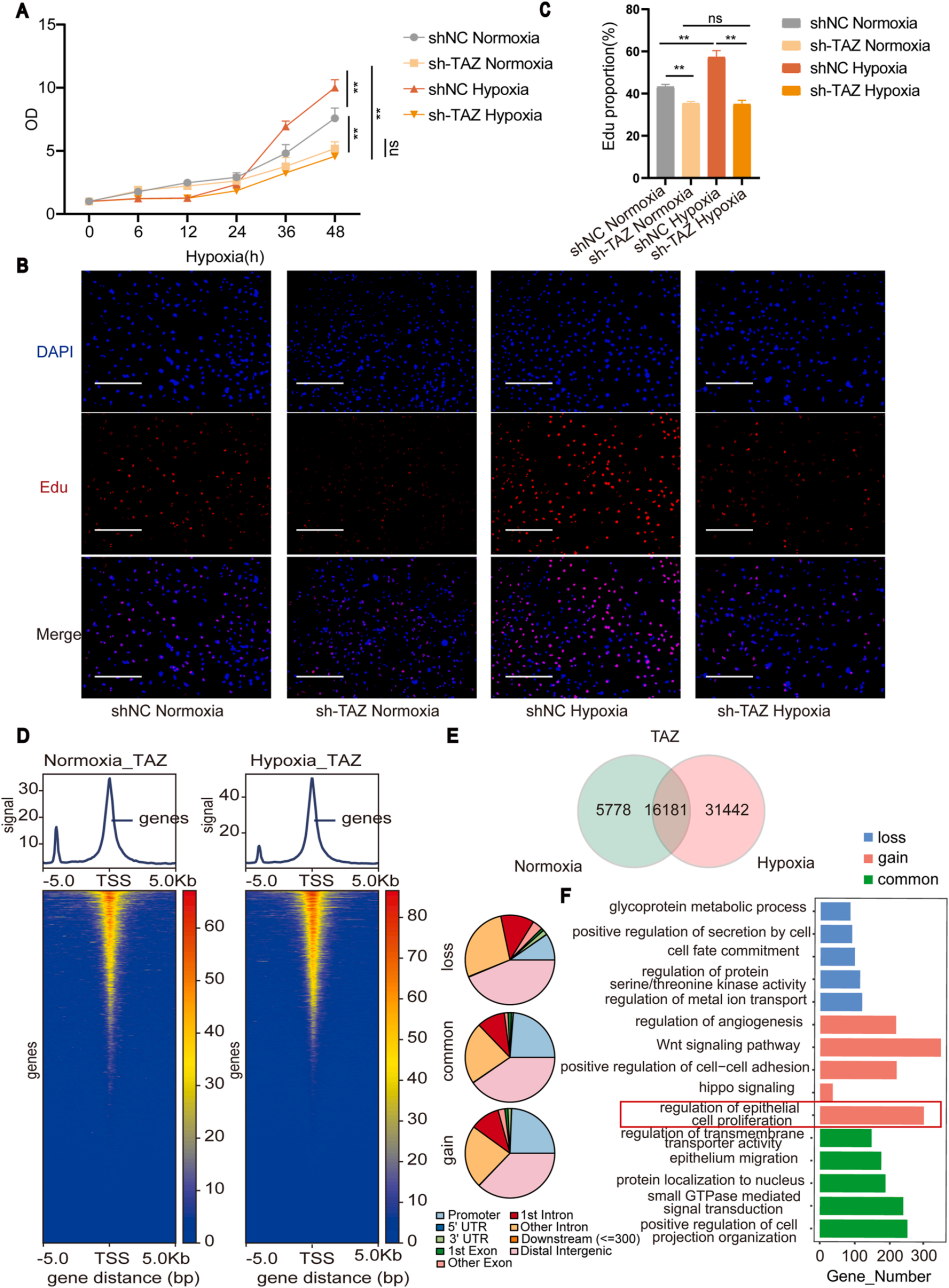
Hypoxia-induced AEC proliferation was mediated by TAZ. **(A)** CCK-8 assay of TC-1 cell proliferation. **(B, C)** Cell proliferation activity evaluated by EdU incorporation assay and quantitative analysis. EdU-positive cells (red) were co-localized with cell nuclei stained by Hoechst 33342 (blue), and the proliferation rate was subjected to quantitative analysis. *n* = 3. **(D)** The CUT&Tag data depicting the enrichment of TAZ at the transcription start site (TSS) in normoxic and hypoxic TC-1 cells. The heatmap consolidated CUT&Tag signals ranging from −5 kb to +5 kb around the TSS center. The pie chart shows the genomic distribution of up-regulated, unchanged, and down-regulated peaks associated with *TAZ* in TC-1 cells. **(E)** The Venn diagram illustrating the number of TAZ peaks that were up-regulated, unchanged, and down-regulated in hypoxic TC-1 cells compared with normoxic conditions. **(F)** Genes associated with these peaks were subjected to GO analysis to identify TAZ-specific functions. TAZ, transcriptional co-activator with PDZ-binding motif; AEC, alveolar epithelial cell; CUT&Tag, cleavage under targets and tagmentation; GO, Gene Ontology.

It has been reported that following hypoxia, AECs undergo self-repair mechanisms to adapt to tissue damage, with enhanced proliferation being a prominent feature.^17^ To elucidate the role of TAZ in this process, we employed CCK8 and EdU assays, which revealed that hypoxia significantly increased the proliferation of AECs. Furthermore, knockdown of TAZ in TC-1 cells markedly inhibited proliferation under both normoxic and hypoxic conditions compared with control TC-1 cells, suggesting that hypoxia promotes the proliferation of AECs via the TAZ pathway (Fig. 3A–C).

It has also been reported that TAZ, functioning as a transcriptional co-activator, modulates the transcription of downstream target genes, a process that is highly dependent on the specific cell type and the stimuli received by the cells.^25^ Given the elevated expression of TAZ in AECs under hypoxic conditions, we conducted CUT&Tag sequencing in TC-1 cells under both normoxic and hypoxic conditions using a TAZ-specific antibody to assess the genomic distribution of TAZ. Under normoxic conditions, 21,959 TAZ-enriched peaks were identified in TC-1 cells, whereas hypoxic stimulation led to the identification of 47,623 TAZ-enriched peaks, indicating a substantial increase in TAZ-enriched genomic regions following hypoxic exposure (Fig. 3D and E). Gene Ontology (GO) analysis of these peaks revealed significant enrichment in signaling pathways associated with epithelial cell proliferation (Fig. 3F). This finding suggests that hypoxic stimulation induces notable alterations in the binding sites of TAZ target genes. Furthermore, the peaks exhibiting changes in TAZ binding under hypoxic conditions were predominantly located in promoter regions, with a marked increase in their enrichment levels (Fig. 3D). Partial gene visualization data are provided in Figure S2A. These results indicate that hypoxia may influence the proliferation of AECs by altering the chromosomal distribution of TAZ.

### TAZ regulates the deposition of H2A.Z on chromatin through interaction with H2A.Z

**Figure 4:**
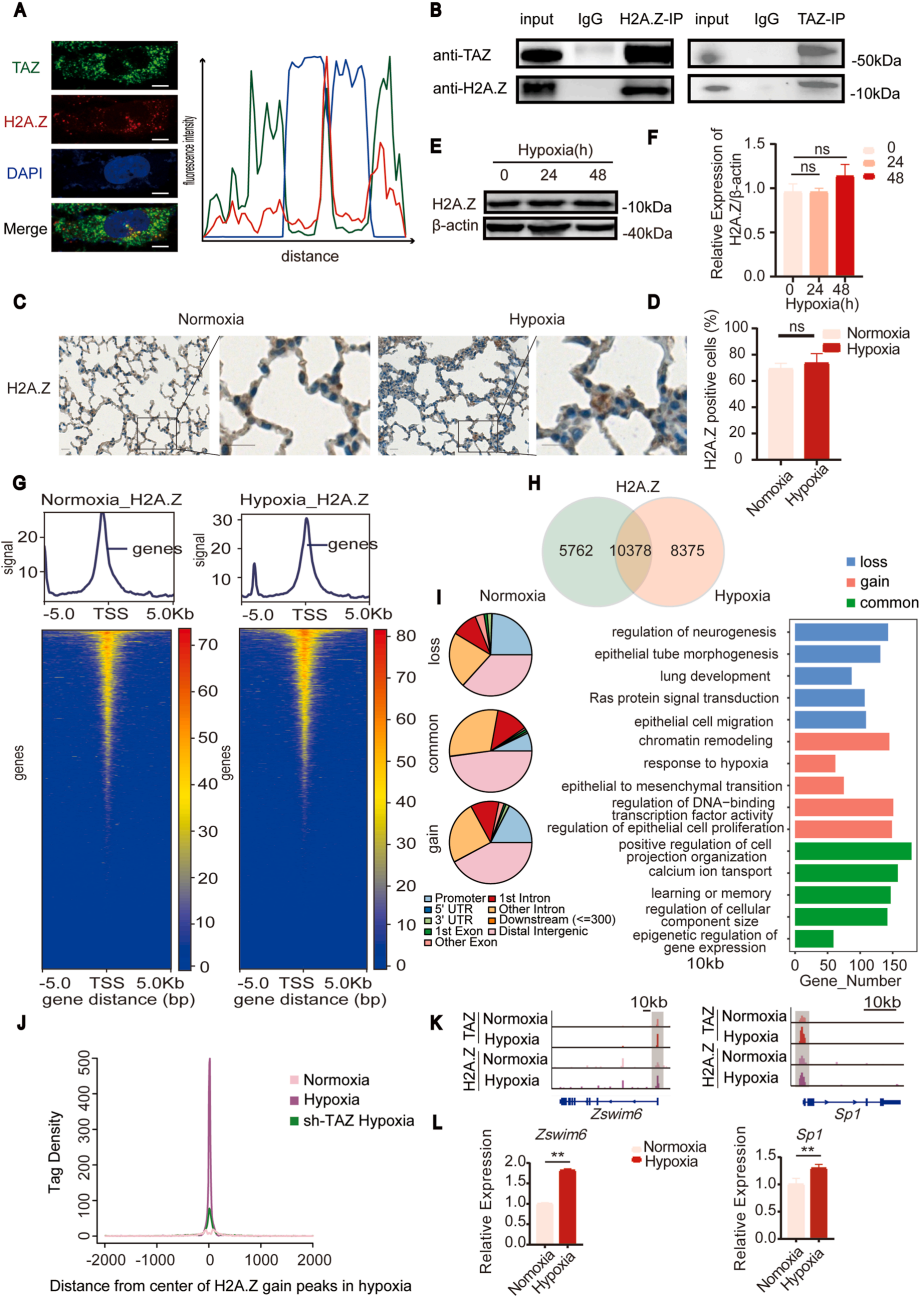
TAZ regulates the deposition of H2A.Z on chromatin through interaction with H2A.Z. **(A)** Co-localization of TAZ (green) and H2A.Z (red) in TC-1 cells, with cell nuclei counterstained by DAPI (blue). **(B)** Co-immunoprecipitation assay between TAZ and H2A.Z in TC-1 cells. **(C)** Immunohistochemical assay of lung tissue sections demonstrated the expression of H2A.Z. Scale bar = 20 μm. **(D)** The bar graph illustrating the quantitative evaluation of H2A.Z expression in representative sections. *n* = 5. **(E, F)** Western blotting analysis and quantification of H2A.Z expression in the mouse alveolar epithelial cell line TC-1 under normoxic and hypoxic conditions. β-actin was used as a loading control. *n* = 3. **(G)** The CUT&Tag data depicting the enrichment of H2A.Z at the transcription start site (TSS) in normoxic and hypoxic TC-1 cells. The heatmap consolidated CUT&Tag signals ranging from −5 kb to +5 kb around the TSS center. The pie chart shows the genomic distribution of peaks associated with H2A.Z in TC-1 cells. **(H)** The Venn diagram illustrating the number of H2A.Z peaks in hypoxic TC-1 cells compared with normoxic conditions. **(I)** Genes associated with these peaks were subjected to GO analysis to identify H2A.Z-specific functions. **(J)** H2A.Z CUT&TAG-seq fragments profiles around the center of H2A.Z gain peaks in hypoxia. **(K)** The IGV data displaying the genomic tracks of TAZ and H2A.Z distribution at the promoters of genes (*Zwsim6* and *Sp1*) in TC-1. **(L)** Relative mRNA expression of *Zwsim6* and *Sp1* in TC-1. *n* = 3. TAZ, transcriptional co-activator with PDZ-binding motif; CUT&Tag, cleavage under targets and tagmentation; GO, Gene Ontology; IGV, integrated genomics viewer; Zswim6, zinc finger SWIM-type containing 6; Sp1, specificity protein 1.

H2A.Z recruits a variety of interacting proteins that play a critical role in the activation or repression of its target genes.^20^ Our previous research indicates the presence of numerous potential interacting proteins of H2A.Z, including TAZ in 293T cells, identified through the bPPI-seq screening method.^22^ To validate the interaction between H2A.Z and TAZ, we initially observed a distinct co-localization of H2A.Z and TAZ within TC-1 cells using immunofluorescence assays, which suggests a potential interaction between these two proteins (Fig. 4A). To further substantiate this finding, we performed Co-IP assays in TC-1 cells and confirmed the interaction between H2A.Z and TAZ, as evidenced by the co-purification of both proteins in the immunoprecipitate (Fig. 4B).

We further observed the lung tissue and TC-1 cells and found that hypoxia did not influence the overall protein levels of H2A.Z (Fig. 4C–F). To investigate whether hypoxia altered the deposition of H2A.Z on target genes, we conducted CUT&Tag sequencing in TC-1 cells. The results showed that normoxic TC-1 cells exhibited 16,140H2A.Z-enriched peaks, whereas hypoxia stimulation led to 183,753H2A.Z-enriched peaks (Fig. 4G and H). We subsequently analyzed the altered genes and performed a GO analysis of the pathways they were enriched in. Our findings indicated that these peaks were primarily enriched in signaling pathways related to chromatin remodeling and cell proliferation (Fig. 4I). Integrated Genomics Viewer (IGV) data revealed hypoxia-induced chromatin remodeling at the promoter regions of selected genes in TC-1 cells (Fig. S2B).

To determine the effect of TAZ on H2A.Z deposition, we acquired midpoint data for H2A.Z gain peaks in TC-1 cells after hypoxia exposure and plotted the distribution of H2A.Z peaks at these loci under both normoxic and hypoxic conditions, with and without TAZ knockdown (Fig. 4J). Our analysis demonstrated a significant increase in H2A.Z peak intensity following hypoxia; however, a marked reduction was observed in the hypoxic group upon TAZ knockdown (Fig. 4J). We then randomly selected two genes (zinc finger SWIM-type containing 6 (*Zswim6*) and specificity protein 1 (*Sp1*)) with increased enrichment of H2A.Z and TAZ on their promoters following hypoxia exposure. Their mRNA expression was examined by quantitative reverse transcription PCR. Results demonstrated that the transcriptional levels of these genes were significantly up-regulated (Fig. 4K and L). These findings indicate that while hypoxia does not influence the overall expression of H2A.Z, it alters the deposition of H2A.Z at target genes to regulate transcription, likely through the interaction between H2A.Z and TAZ.

### Hypoxia up-regulates IL-6 expression through modulating TAZ-dependent H2A.Z deposition in the IL6 gene locus

**Figure 5:**
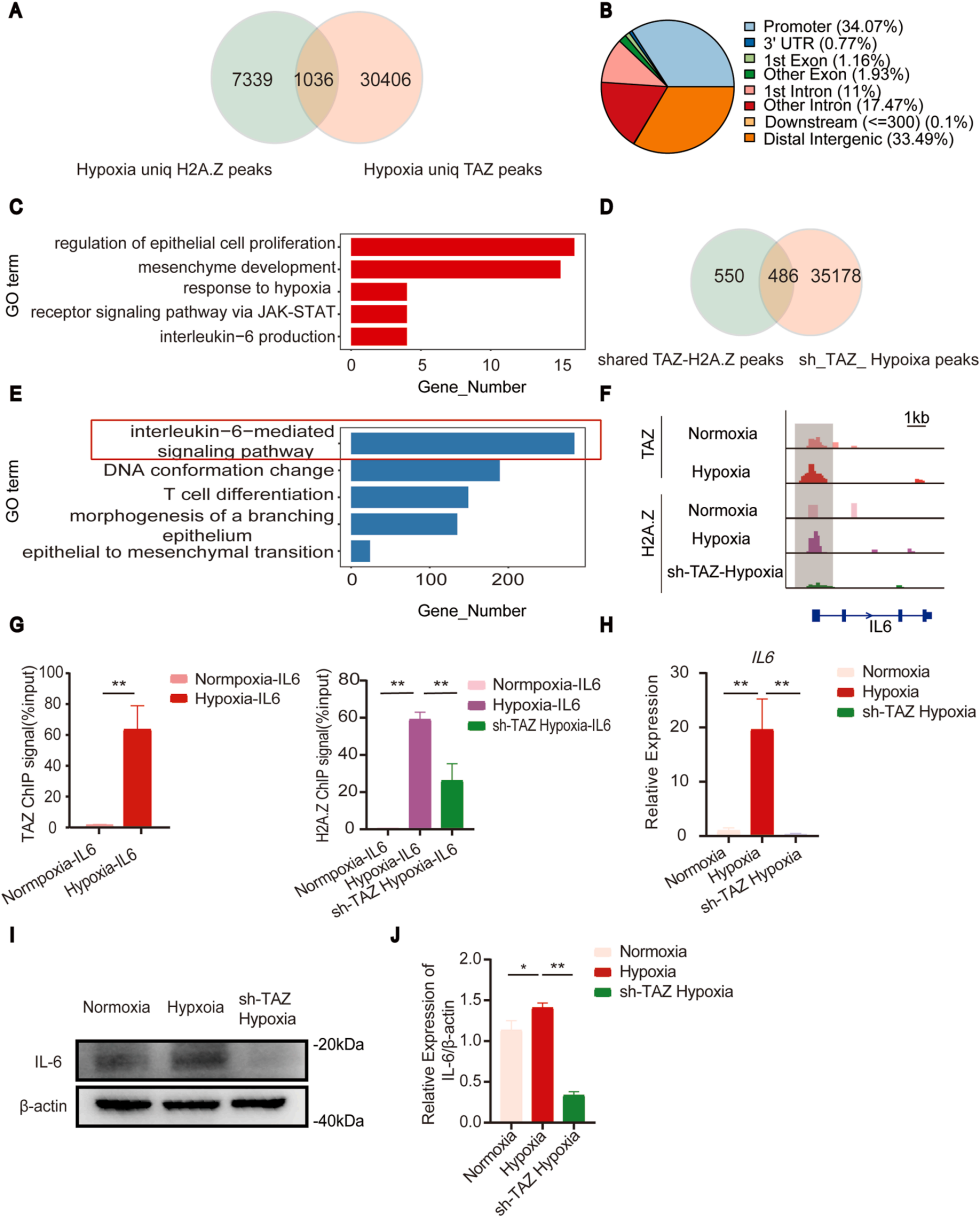
Hypoxia up-regulates *IL6* expression through TAZ-dependent H2A.Z deposition in the *IL6* gene locus. **(A)** The Venn diagram displaying the number of up-regulated peaks for TAZ and H2A.Z in hypoxic TC-1 cells. **(B)** The pie chart illustrating the genomic distribution of up-regulated peaks for TAZ and H2A.Z in hypoxic TC-1 cells. **(C)** GO analysis of peaks that were up-regulated for both *TAZ* and H2A.Z in TC-1 cells, as determined by CUT&Tag analysis. **(D)** The Venn diagram highlighting the differential peaks between up-regulated peaks for both *TAZ* and H2A.Z in hypoxic TC-1 cells and H2A.Z-enriched peaks in TC-1 cells of stable *TAZ* knockdown after hypoxia. **(E)** GO analysis of differential peaks between up-regulated peaks for both *TAZ* and H2A.Z in hypoxic TC-1 cells and H2A.Z-enriched peaks in TC-1 cells of stable *TAZ* knockdown after hypoxia, as determined by CUT&Tag analysis. **(F)** Genomic track shows the distribution of TAZ and H2A.Z at the *IL6* promoter in normoxic, hypoxic, and *TAZ*-knockdown/hypoxic TC-1 cells. **(G)** Chromatin immunoprecipitation-quantitative PCR analysis of the binding levels of TAZ and H2A.Z to the *IL6* promoter in normoxic and hypoxic TC-1 cells, and of H2A.Z to the *IL6* promoter in *TAZ*-knockdown/hypoxic TC-1 cells. Chromatin immunoprecipitation signals were normalized to input. **(H)** Relative mRNA expression of *IL6* in normoxic, hypoxic, and *TAZ-*knockdown/hypoxic TC-1 cells. **(I, J)** Western blotting analysis and quantification of *IL6* expression in normoxic, hypoxic, and *TAZ*-knockdown/hypoxic TC-1 cells. β-actin is used as a loading control. *n* = 3. TAZ, transcriptional co-activator with PDZ-binding motif; GO, Gene Ontology; CUT&Tag, cleavage under targets and tagmentation; IL6, interleukin 6.

To ascertain the impact of TAZ on H2A.Z localization at target genes under hypoxic conditions, we analyzed the genomic peaks for both H2A.Z and TAZ. By intersecting the 8375H2A.Z peaks and the 31,442 TAZ peaks following hypoxic stimulation, which were greater than those observed under normoxic conditions, we identified 1036 common elevated peaks for both H2A.Z and TAZ in hypoxic AECs (Fig. 5A). These peaks were significantly enriched at the promoter regions (Fig. 5B). GO analysis of these up-regulated genes revealed significant enrichment in pathways related to cell proliferation (Fig. 5C). Given the increased expression of TAZ in hypoxic AECs, we performed CUT&Tag sequencing in TAZ-knockdown TC-1 cells under hypoxic conditions using an H2A.Z antibody and intersected the results with the 1036 elevated peaks. This analysis identified 550 peaks where H2A.Z was significantly altered and regulated by TAZ (Fig. 5D). GO analysis of the 550 peaks revealed a notable predominance of pathways associated with IL-6 mediation, highlighting this pathway as the most enriched within our dataset (Fig. 5E). This enrichment underscores the pivotal role of IL-6 signaling in the biological processes influenced by these peaks. Using the IGV, we observed increased enrichment of TAZ and H2A.Z at the *IL6* promoter under hypoxic conditions, which was reduced upon TAZ knockdown (Fig. 5F). Consistent with the CUT&Tag results, ChIP-quantitative PCR analysis confirmed increased enrichment of H2A.Z and TAZ at the *IL6* promoter under hypoxic conditions, and this enrichment was diminished upon TAZ knockdown (Fig. 5G). The quantitative reverse transcription PCR and Western blotting analyses of *IL6* expression in these three conditions revealed increased *IL6* expression under hypoxic conditions, which was attenuated by TAZ knockdown (Fig. 5H–J). These findings suggest that hypoxia directly regulates *IL6* transcription through the H2A.Z-TAZ axis in AECs.

### Hypoxia-induced IL-6 expression in AECs promotes M2 polarization

**Figure 6:**
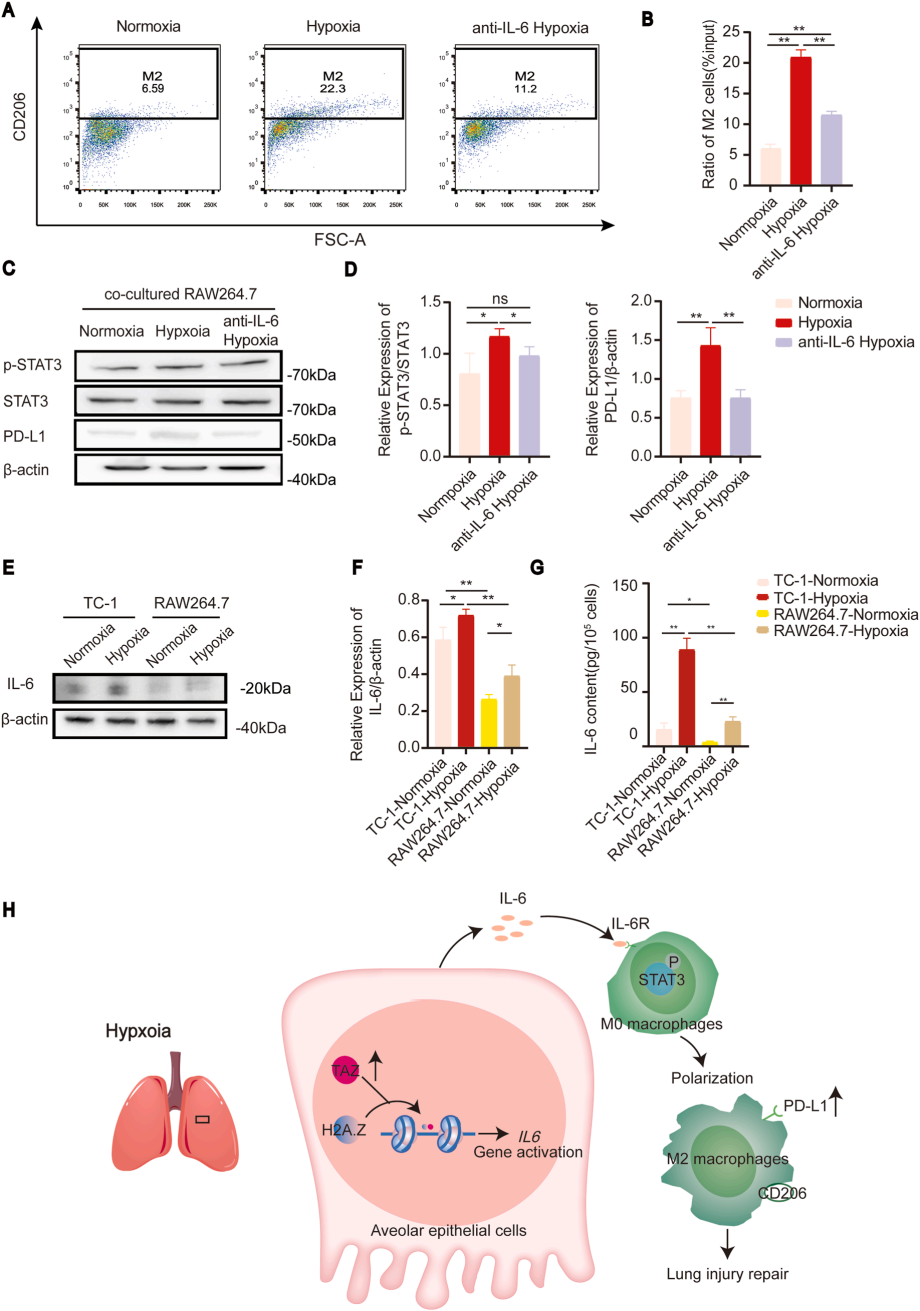
Hypoxia-induced *IL6* expression in AECs promotes M2 Polarization. **(A, B)** Flow cytometry analysis of the proportion of CD206^+^ (M2) macrophages (F4/80+) in co-cultures with TC-1 cells. The ratio of M2 macrophages in RAW264.7 cells is depicted. **(C, D)** Western blotting analysis and quantification of the expression levels of p-STAT3, STAT3, and PD-L1 in RAW264.7 cells co-cultured with TC-1 cells. β-actin is used as a loading control. *n* = 3. **(E, F)** Western blotting analysis and quantification of IL-6 expression levels in both TC-1 cells and RAW264.7 cells. *n* = 3. **(G)** ELISA analysis of the IL-6 expression levels in both TC-1 and RAW264.7 cells. *n* = 3. **(H)** Schematic diagram of the mechanism for how TAZ induces IL-6 production and M2 polarization by affecting H2A.Z chromatin deposition under hypoxic conditions. IL6, interleukin 6; AEC, alveolar epithelial cell; TAZ, transcriptional co-activator with PDZ-binding motif; STAT3, signal transducer and activator of transcription 3; PD-L1, programmed cell death ligand 1; ELISA, enzyme-linked immunosorbent assay.

To examine the impact of hypoxia-induced up-regulation of *IL6* expression via the TAZ-H2A.*Z* axis, we conducted co-culture experiments with AECs and macrophages. Flow cytometry analysis demonstrated that hypoxic co-cultures exhibited a more pronounced polarization of macrophages towards the M2 phenotype compared with normoxic co-cultures. The introduction of an IL-6 antagonist into the hypoxic co-culture medium attenuated M2 polarization, highlighting the critical role of IL-6 in this process (Fig. 6A and B). Prior research has established that IL-6 can drive M2 polarization in macrophages through the activation of STAT3, resulting in elevated PD-L1 production.^26^ It has been confirmed that PD-L1, produced by M2 macrophages, facilitates repair following lung injury.^27^ Western blotting analysis of macrophages under hypoxic conditions revealed increased levels of p-STAT3 and PD-L1 in hypoxic co-cultures, which were diminished upon the addition of the IL-6 antagonist (Fig. 6C and D). These findings suggest that the repair process following lung injury under hypoxic conditions may be mediated by IL-6 through STAT3 activation, promoting M2 polarization and enhanced PD-L1 production.

To ascertain the source of IL-6, Western blotting analysis and ELISA were employed to evaluate IL-6 protein expression in TC-1 and RAW264.7 under both normoxic and hypoxic conditions. The results demonstrated that AECs secreted significantly higher levels of IL-6 compared with macrophages under normoxic conditions, and this disparity was markedly amplified under hypoxic conditions, with AECs serving as the predominant producers of IL-6 under hypoxia (Fig. 6E–G). These observations suggest that hypoxia up-regulates IL-6 expression in AECs through the TAZ-H2A.Z pathway, thereby facilitating macrophage M2 polarization (Fig. 6H).

## Discussion

Hypoxia, a common cause of lung injury, has been documented to elicit inflammatory cell infiltration, alveolar septal thickening, and alveolar collapse in lung tissue.^2^ Nonetheless, existing interventions to mitigate hypoxia-induced lung injury remain limited, underscoring the necessity for further investigation into the mechanisms governing lung repair post-hypoxia injury. To elucidate the effects of hypoxia on lung injury in mice, we utilized a hypoxia chamber to replicate high-altitude conditions. In contrast to methodologies employing chemical agents such as sodium nitrite or cobalt chloride to mimic hypoxia, or substances that bind to hemoglobin and diminish oxygen-carrying capacity, our method more accurately simulates the low-oxygen environment characteristic of high-altitude regions. Additionally, through the application of our previously established bPPI-seq technology, we identified an interaction between H2A.Z and TAZ, which was subsequently confirmed via Co-IP. This interaction exhibits greater specificity compared with techniques such as yeast two-hybrid (Y2H), which are notorious for their high false-positive rates and reduced specificity. This study predominantly employed CUT&Tag analysis and experimental approaches to examine critical transcriptional regulators implicated in lung repair following hypoxia-induced injury, thereby offering potential targets for novel therapeutic strategies against hypoxia-induced lung injury.

During the process of hypoxia-induced lung injury, macrophage polarization plays a critical role.^28^ Our findings are consistent with existing literature, demonstrating elevated levels of M1 and M2 polarization in mouse lung tissue following hypoxia, as evidenced by Western blotting analysis of the markers iNOS and ARG1.^28^^,^^29^ Specifically, during the early stages of hypoxia, we observed exacerbated lung injury, with the iNOS/ARG1 ratio peaking at 4 days, which is likely closely associated with the activation and predominance of M1 macrophages. These cells initiate and amplify local inflammatory responses, contributing to increased lung injury. However, by day 14 of hypoxia, a reduction in lung injury was noted. This observation, supported by our analysis of the iNOS/ARG1 ratio, indicates a decline in the ratio, suggesting a shift toward M2 macrophage polarization. This shift may represent an adaptive response to hypoxia stress, potentially involving a transition from M1 to M2 macrophages, thereby mitigating further inflammation and tissue damage. To further validate the role of M2 macrophages in hypoxia-induced lung injury, we conducted M2 macrophage transfer experiments. Hematoxylin-eosin staining and BALF protein concentration measurements indicate that M2 macrophages can protect against hypoxia-induced lung injury, which is consistent with previous reports.^24^

Under hypoxic conditions, it has been reported that the protein expression level of TAZ is significantly elevated.^14^^,^^15^ Furthermore, evidence indicates that TAZ protein in AECs plays a crucial role in the repair process following lung injury, exhibiting anti-inflammatory effects post-injury and promoting the proliferation and differentiation of AECs.^16^, ^17^, ^18^ This suggests a potentially significant role for TAZ in cellular adaptation to hypoxic conditions and the facilitation of damage repair. However, the mechanisms by which the increased expression of TAZ post-hypoxia promotes repair remain unclear. Our study has demonstrated that M2 macrophages can facilitate repair. Therefore, in this study, we focused on the increase in TAZ expression in AECs post-hypoxia, which promotes macrophage polarization towards the M2 phenotype, possibly as a stress response mechanism. However, this polarization shift was insufficient to reverse tissue damage caused by M1 macrophages in the early stages of hypoxia.

Studies have demonstrated that AECs, particularly AT2, play a pivotal role in the repair process following lung injury. AT2 cells serve not only as progenitor cells within the alveolar epithelium but also exhibit robust proliferative and differentiation capabilities. Following injury, these cells can self-amplify and differentiate into AT1, thereby reconstructing the alveolar architecture and restoring gas exchange function.^30^ Moreover, AT2 cells contribute to reducing alveolar fluid accumulation by clearing fluid from the alveoli and secreting surfactant, which enhances oxygenation.^31^ Literature has reported that in the bleomycin-induced lung injury model, TAZ expression in AT2 cells progressively increases with the duration of lung injury, promoting post-injury repair. In contrast, TAZ levels in AT1 cells remain unchanged.^16^ In this study, we found that TAZ is expressed in both AT1 and AT2 cells, however, its expression significantly increases in AT2 cells following hypoxic injury, whereas no significant change is observed in AT1 cells after hypoxia. Our results are consistent with the reports in relevant literature, suggesting that TAZ mainly plays a key role in AT2 cells during the repair process of lung injury.

Our research team previously identified H2A.Z as an interactor of TAZ,^22^ a finding that we have further validated in this study. In line with existing literature,^19^ our observations confirm that TAZ plays a crucial role in maintaining H2A.Z enrichment at target gene loci. To explore the impact of TAZ on H2A.Z localization under hypoxic conditions, we identified peaks where both TAZ and H2A.Z levels were elevated. Given the increased expression of TAZ following hypoxia, we performed CUT&Tag sequencing in TAZ-knockdown TC-1 cells under hypoxic conditions using an H2A.Z antibody and compared the results with the previously identified 1036 peaks. This analysis revealed 550 peaks where H2A.Z enrichment was significantly altered and regulated by TAZ. GO analysis indicated that the IL-6 signaling pathway was the most significantly enriched. Previous studies have shown that TAZ promotes IL-6 transcription, which is a critical factor in the progression of colorectal tumors.^32^ Another study has demonstrated that IL-6, a downstream effector of TAZ, is essential for maintaining the stem-like characteristics of breast cancer cells.^33^

In this study, TAZ plays a critical role in maintaining H2A.Z enrichment at the *IL6* promoter; knockdown of TAZ reduces H2A.Z enrichment to levels observed under normoxic conditions. This suggests that TAZ may enhance *IL6* transcriptional activity by promoting H2A.Z recruitment or stabilization, thereby contributing to inflammation and repair following lung injury. Notably, H2A.Z has been reported to regulate oncogenic transcription by depositing at the promoters of TAZ target genes during cancer progression, tumor heterogeneity, and potential epigenetic changes.^19^ Our study extends this concept, indicating that similar epigenetic modifications occur in hypoxia-induced lung injury. These findings not only deepen our understanding of lung repair mechanisms post-injury but also identify potential molecular targets for the development of novel therapeutic strategies for lung injury and associated diseases. Recent studies have demonstrated that under hypoxic conditions, the hypoxia inducible factor 1 subunit alpha (HIF-1α)-TAZ complex enhances TAZ expression and interaction,^15^ likely participating in the regulation of *IL6* transcription. Further research is required to elucidate the detailed mechanisms of the HIF-1α-TAZ-H2A.Z complex in lung injury repair. Additionally, as our study was primarily conducted at the cellular level, validation in animal models and clinical samples is essential.

Our findings indicate that TAZ can modulate the deposition of H2A.Z on chromatin, thereby affecting gene transcription changes in AECs following hypoxia. However, the exact mechanisms through which TAZ regulates H2A.Z deposition warrant further exploration. Notably, a recent study has demonstrated that in the context of oncogene transcriptional regulation, TAZ interacts with the SRCAP complex to facilitate H2A.Z deposition at target loci.^19^ Despite our inability to directly confirm TAZ’s interaction with the SRCAP complex in AECs due to the unavailability of specific antibodies for the murine SRCAP complex, this interaction represents a plausible mechanism that we intend to investigate further to elucidate the precise regulatory role of TAZ in H2A.Z deposition on chromatin.

In line with our observations, a considerable body of prior research has demonstrated that IL-6 can facilitate macrophage polarization towards the M2 phenotype.^26^^,^^34^ For example, following cardiac injury, cardiomyocytes and adjacent cells continuously release IL-6, which promotes M2 macrophage polarization.^34^ Additionally, tumor-associated adipocytes secrete substantial quantities of IL-6, leading to M2 macrophage polarization via STAT3 activation.^26^ Our findings suggest that hypoxia induces TAZ expression in AECs, which enhances IL-6 transcription, thereby promoting M2 macrophage polarization in the lung. We have identified that these mechanisms may similarly occur in AECs. Under hypoxic conditions, co-cultured macrophages exhibit increased expression of Stat3 and PD-L1, which is attenuated by the addition of an IL-6 antagonist. These results corroborate the mechanism by which IL-6 promotes M2 macrophage polarization through STAT3 activation, and the PD-L1 produced by M2 macrophages plays a crucial role in lung injury repair. Furthermore, existing studies have shown that rat alveolar type II (ATII) cells *in vivo* can produce a significant amount of IL-6, with spontaneous secretion levels five times higher than those of alveolar macrophages.^35^ This observation aligns with our experimental results, indicating that AECs produce IL-6 more efficiently than macrophages under hypoxic conditions.

Although this study has achieved certain advancements in elucidating the molecular mechanisms underlying hypoxia-induced lung injury and M2 macrophage polarization, one notable limitation lies in its heavy reliance on the RAW264.7 macrophage cell line. While RAW264.7 cells have been extensively utilized as a model system for investigating macrophage polarization dynamics,^36^^,^^37^ they may incompletely recapitulate the complexity and heterogeneity of primary macrophages under *in vivo* conditions. Furthermore, inherent discrepancies exist between *in vitro* culture environments and the *in vivo* milieu, where the cultured cell lines may not fully recapitulate the intricate physiological and pathological processes occurring *in vivo*. Of relevance to pulmonary pathology, emerging evidence highlights substantial heterogeneity among macrophage subsets within lung tissue. Due to current experimental constraints, our investigation was confined to the classical M1-M2 dichotomy. To address this gap, we plan to conduct further investigations to systematically characterize the effects of hypoxic alveolar epithelial cells on diverse pulmonary macrophage subsets, including tissue-resident macrophages and monocyte-derived populations, in future research endeavors.

In conclusion, our findings demonstrate that TAZ interacts with H2A.Z in AECs, facilitating the deposition of H2A.Z at the *IL6* gene promoter under hypoxic conditions, which in turn regulates *IL6* transcription. Furthermore, we have verified that AECs secrete IL-6 in response to hypoxic stimuli, thereby promoting macrophage polarization toward the M2 phenotype. This study elucidates a novel mechanism involving TAZ and H2A.Z in AECs during lung injury repair, highlighting the potential significance of IL-6 in modulating macrophage polarization and lung tissue regeneration. These results offer promising avenues for future research and therapeutic strategies. Additional investigations are warranted to delineate the precise roles of TAZ and H2A.Z in lung injury repair and to explore their functions in other cell types and disease settings.

## CRediT authorship contribution statement

**Paiyu Liu:** Writing – original draft, Data curation, Conceptualization. **Mengjie Zhang:** Writing – original draft, Investigation, Funding acquisition, Data curation. **Jitao Zeng:** Writing – review & editing, Visualization, Formal analysis. **Yafei Gao:** Investigation. **Xiaochong He:** Investigation. **Wenying Liu:** Investigation. **Peng Wang:** Formal analysis. **Bing Ni:** Writing – review & editing, Writing – original draft, Methodology, Investigation, Funding acquisition, Conceptualization.

## Data availability

The raw sequence data presented in this paper have been deposited in the Genome Sequence Archive (GSA)^38^ hosted by the National Genomics Data Center (NGDC),^39^ China National Center for Bioinformation/Beijing Institute of Genomics, Chinese Academy of Sciences (Accession Number: CRA020874). The data are publicly accessible at https://ngdc.cncb.ac.cn/gsa. Any additional information necessary for reanalyzing the data reported in this paper is available upon request from the corresponding author. This paper does not include any original code.

## Funding

This work was financially supported by the General Project of the 10.13039/501100002865Chongqing Science and Technology Bureau (China) (No. CSTB2024NSCQ-MSX0838).

## Conflict of interests

The authors declared no conflict of interests.
