# Supplementary material for: Co-localization of TAZ and H2A.Z up-regulates IL-6 expression in alveolar epithelial cells to polarize M2 cells to alleviate hypoxia-induced lung injury
Source: Genes Dis. 2025 Jun 9;13(6):101713. doi: 10.1016/j.gendis.2025.101713 (PMC13485503; doi:10.1016/j.gendis.2025.101713)
Supplement: Supplementary file 1 [file mmc1.docx]

**Supplementary Figures**

**Supplementary Figure 1. In Vivo Tracking of IL-4-Polarized M2 Macrophages in Mice.**

(A) Flow cytometry analysis of the proportion of CD206^+^ (M2) macrophages (F4/80^+^). The ratio of M2 macrophages in BMDM stimulated with PBS and IL-4 is presented. (B) IF images of lung tissue after intravenous injection of M2 macrophages labeled for tracking.

**Supplementary Figure 2. IGV Data Revealing Hypoxia-Induced Chromatin Remodeling at Promoter Regions of the Selected Genes in TC-1 Cells.**

(A) IGV data displaying the genomic tracks of TAZ distribution at the promoters of genes with up-regulated, unchanged, and down-regulated TAZ peaks induced by hypoxia in TC-1 cells. (B) IGV data displaying the genomic tracks of H2A.Z distribution at the promoters of genes with up-regulated, unchanged, and down-regulated H2A.Z peaks induced by hypoxia in TC-1 cells.
